# Early wound healing outcomes after regenerative periodontal surgery with enamel matrix derivatives or guided tissue regeneration: a systematic review

**DOI:** 10.1186/s12903-019-0766-9

**Published:** 2019-05-07

**Authors:** M. A. Rojas, L. Marini, A. Pilloni, P. Sahrmann

**Affiliations:** 1 Section of Periodontics, Department of Oral and Maxillofacial Sciences, “Sapienza” University of Rome, 00161 Rome, Italy; 2 Clinic of Preventive Dentistry, Periodontology and Cariology, Center of Dental Medicine, University of Zurich, 8032 Zurich, Switzerland

**Keywords:** Periodontal diseases, Periodontal healing, Guided tissue regeneration, Enamel matrix proteins

## Abstract

**Background:**

Proper wound healing after regenerative surgical procedures is an essential issue for clinical success. Guided tissue regeneration (GTR) and application of enamel matrix derivatives (EMD) are common means to regenerate periodontal tissues. Both methods bear considerable advantages due to their special characteristics, but also go along with certain disadvantages. Today, there is no consensus in the literature whether GTR or EMD show better results regarding early wound healing, which is considered a crucial stage in periodontal regeneration. Therefore, the aim of the present systematic review was to compare the early wound healing after regenerative periodontal surgery with either EMD or GTR treatment.

**Methods:**

An electronic literature search in PubMed was performed to identify randomized clinical trials (RCTs) or clinical trials (CTs) comparing regenerative surgery employing EMD and/or GTR in patients with chronic periodontitis. Among the finally included studies, a qualitative and quantitative data extraction regarding early wound healing parameters was performed. Primary outcome parameters were early wound healing index (EWH), flap dehiscence, membrane exposure, suppuration and abscess formation during the first 6 weeks. As secondary parameters, swelling and allergic reactions were assessed.

**Results:**

Seven studies reporting 220 intrabony periodontal defects in 199 patients were analysed.

Flap dehiscence was observed in two studies in 12% of the GTR treated sites and in 10.3% of those treated with EMD. Membrane exposure was evaluated in five studies and was registered in the 28.8% of the defects, while no dehiscence was reported on the EMD group. Swelling was reported only in one study in 8/16 GTR sites and 7/16 EMD sites. Due to considerable heterogeneity of parameters no meta-analysis was possible.

**Conclusions:**

Due to considerable heterogeneity of the published studies a clear beneficial effect of the EMD on the early wound healing outcomes after surgical treatment of periodontal intrabony defects cannot be confirmed.

Standardized RCT studies are needed in order to allow for proper comparison of early wound healing after both types of surgical approaches.

## Background

The World Workshop of the Classification of Periodontal and Peri-Implant Diseases and Conditions of 2017 defines periodontitis as a chronic multifactorial inflammatory disease associated with dysbiotic plaque biofilms and characterized by progressive destruction of the tooth-supporting apparatus [1].

Treatment of periodontitis aims on one hand at preventing further disease progression by minimizing inflammation by active therapy and – on the other hand - at supporting patients in maintaining a healthy periodontium [2].

The management of chronic periodontal disease requires a combination of different therapeutic steps. In first place, a non-surgical approach that includes oral hygiene instructions [3], control of local [4] and systemic factors [5] like the adjustment of excessive forces on single teeth [6] or an untreated diabetes mellitus [7], respectively. Then, supra and subgingival instrumentation is performed as the core step in order to mechanically remove biofilms and mineralized deposits [8, 9]. The latter may be supported by topically or systemically applied pharmacotherapy [10]. In second place, after an adequate healing period, surgical approaches may be indicated to eliminate residual pockets and to create a gingival morphology that allows for efficient plaque control [2]. Likewise, lost tissues might get regenerated by special surgical methods if anatomy and patient characteristics allow for it [11]. It is the aim of such interventions to rebuild each of the tooth-supporting structures, including root cementum, periodontal ligament, and alveolar bone, that were lost due to periodontal inflammation [12, 13].

True regeneration has scientifically been proven especially after conventional guided tissue regeneration (GTR) [14] or the use of enamel matrix derivatives (EMD) [15].

The principles of GTR are based on the exclusion of the proliferating epithelium during the first phase of wound healing. Using a cell-dense membrane, space is provided for slow-proliferating bone and root cementum [14]. On the other hand, EMD, consisting of a heterogeneous mixture of porcine amelogenines, and propylene glycol alginate (PGA) as carrier allows for a pharmacologically induced regeneration of periodontal tissues [16, 17].

Especially in regenerative surgical procedures, early and safe wound closure is a crucial factor for success [18]. This depends on the maintenance of wound stability in the first post-surgical weeks [19, 20]. Particularly, the critical significance of primary intention healing for periodontal regeneration has been demonstrated in a retrospective study on GTR procedures by Trombelli et al. showing significant lower values of bone level gain when the membrane got previously exposed [21].

Several surrogate parameters are used to describe early wound healing in oral soft tissues. Special scores for early periodontal wounds have been proposed in order to comprehensively describe healing by numerous surrogate parameters like tissue colour, bleeding, characteristics of incision margins and presence of suppuration [22], assessment of wound closure, abscess formation, fibrin and necrosis [23] and, furthermore, edema, erythema, suppuration, patient discomfort and flap dehiscence [24]. Recently, the Early Wound Healing Score (EHS) was introduced to assess wound healing by primary intention 24 h post-surgery through the evaluation of clinical signs of re-epithelialization, haemostasis and inflammation [25]. However, most clinical studies report early complications reflecting on wound dehiscence and post-operative pain only [26, 27].

Success of regenerative therapy, however, is multi-factorial and depends on numerous aspects. The placement of membranes has - besides the intended beneficial impact - a potential side effect that can hamper the surgical outcome [28–30]. Since the cell-dense membrane does not only hamper cell migration but also diffusion, the nutrition of the gingival tissues is limited and may tend to result in wound dehiscence and membrane exposure. As a consequence, membrane surfaces get colonized by oral biofilm which, in turn, leads to further inflammation, jeopardizing the success of the surgical procedure [29, 30].

The use of EMD on the other hand, has been described to have a positive effect on early wound healing. Specifically, EMD has been shown to accelerate reepithelialization, wound closure, resolution of inflammation and prolonged blood vessels formation [31–33].

Indeed, in some studies more post-surgical complications following GTR than after EMD application have been reported [15, 28] whereas others found no differences in the healing process [26, 27].

So far there is no consensus whether the use of EMD may show better early wound healing as compared to GTR.

Therefore, this systematic review aimed at comparing early wound healing after regenerative periodontal surgery with GTR or EMD application. Our hypothesis was that there is beneficial effect of the EMD when compared to GTR on the early wound healing after surgical treatment of periodontal intrabony defects.

## Methods

The Preferred Reporting Items for Systematic Reviews and Meta-Analyses (PRISMA) statement was consulted to the process of the present systematic review.

### Focused question

In periodontal defects, are the early wound healing outcomes after periodontal regenerative surgery better after the use of EMD as compared to GTR?

### Eligibility criteria

The studies were selected according to the following criteria:

#### Inclusion criteria

- Randomized Clinical Trials (RCTs) or Clinical Trials (CTs) comparing surgical regenerative interventions using Enamel Matrix Derivatives or Membranes, both in combination or without bone substitutes in the surgical treatment of intrabony periodontal defects or furcation involvement defects;
- Human adults (> 30 years) with chronic periodontitis and good general health status;
- Non-smoker patients;
- Generally healthy patients.

#### Exclusion criteria

- Non adult patients;
- Systemic diseases;
- Patients with aggressive periodontitis;
- Smokers

The outcome was assessed in terms of early wound healing during the time period of one to six weeks. Primary outcome parameters were early wound healing index (EWH), flap dehiscence, membrane exposure (in GTR group), suppuration and abscess. As secondary parameters, swelling and allergic reactions were used.

### Search strategy

A comprehensive and systematic electronic search of US National Library of Medicine (Pubmed) was performed. The search was conducted for trials in the period up to July 2018. The following key words were used: (guided tissue regeneration OR regenerative OR Emdogain OR enamel matrix derivatives OR amelogenin OR membrane) AND (periodontitis OR periodontal therapy OR periodontal surgery) AND (RCT OR clinical trial).

The literature research was performed without language restrictions.

### Selection of the studies

Previous to the screening process, the first 50 titles and abstracts were used to calibrate the two reviewers (MR and LM) with a senior researcher (PS). Consequently, two reviewers (MR and LM) screened independently all titles and abstracts. Then, studies potentially complying with the inclusion criteria were selected for full text assessment. After independent assessment, any disagreement between both reviewers was resolved by discussion with a third reviewer (PS).

### Data extraction

Relevant data, including population characteristics, intervention sites characteristics, description of the treatment prior to and at completion of the interventions, post-surgical indications and medications, time of the study, maintenance therapy characteristics and early wound healing parameters assessed were independently extracted by two reviewers (MR and LM).

### Quality assessment of included studies

Following the guidelines of the Cochrane Collaboration [34] a quality assessment of the included studies was performed independently by MR and LM.

Therefore, six domains were evaluated: 1) sequence generation, 2) allocation concealment, 3) blinding of participants and outcome assessors, 4) incomplete outcome data, 5) selective outcome reporting, 6) other sources of bias. In each assessment tool previously mentioned, a judgement of “Yes” or “No” indicated low and high risk of bias, respectively; whereas “Unclear” judgement indicated uncertain risk of bias.

A study was assigned as “Low risk of bias” when all the domains were of low risk of bias. However, when one or more key domains resulted with unclear or high risk of bias, the study was assigned as “Unclear or High risk of bias”.

The quality of non-randomized clinical trials was assessed using the Cochrane Collaboration tool -ROBINS-I tool- (“Risk Of Bias In Non-randomized Studies - of Interventions”) [35, 36]. Seven domains were evaluated: pre-intervention: 1) bias due to confounding, 2) bias in selection of participants into the study; at intervention: 3) bias in classification of interventions; post- intervention: 4) bias due to deviations from intended interventions, 5) bias due to missing data, 6) bias in measurement of outcomes, 7) bias in selection of the reported result. The bias of the studies was assigned as follows:

- “Low risk of bias”: all key domains were of low risk of bias;
- “Moderate risk of bias”: low or moderate risk of bias for all the domains, and moderate risk of bias in any domain;
- “Serious risk of bias”: at least one domain with serious risk of bias but not any critical risk of bias in any domain;
- “Critical risk of bias”: at least one domain with serious critical of bias.

Any disagreement for data extraction and quality assessment was discussed and resolved by consensus. A third reviewer (PS) was consulted when necessary.

## Results

### Search and screening

**Fig. 1:**
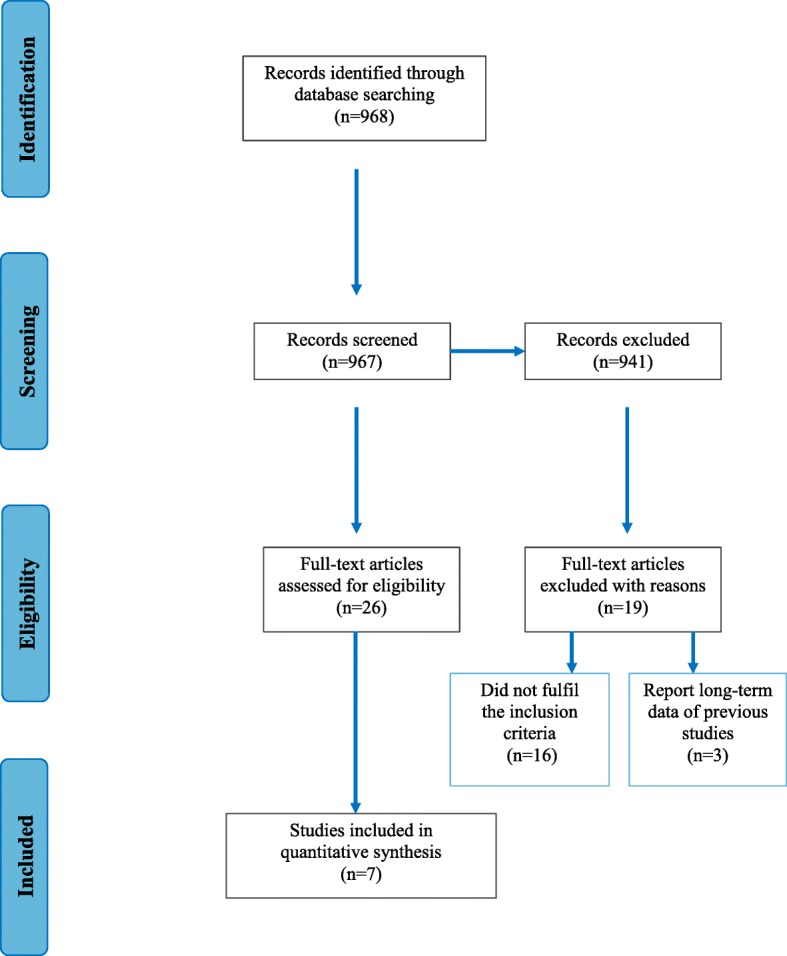
Flow diagram (PRISMA format) of the screening and selection process

The search strategy generated 968 potentially fitting articles. After title and abstract screening, 26 articles were eligible for possible inclusion (Fig. 1). During full-text assessment, nineteen articles were excluded due to smoking (nine articles) [37–45], patients younger than 30 years of age (three studies) [46–48], diagnosis of aggressive periodontitis (two studies) [49, 50], missing outcomes for early wound healing evaluation (one study) [51] and not-fitting treatments group (one study) [52]. Moreover, three studies [53–55] were found to report long-term results of already included publications and were likewise excluded. Finally, seven studies were included [56–62]. Reviewer agreement for title and abstract screening was 92% and agreement for the full text screening before discussion was 88%(Table 1).

**Table 1:** Excluded studies

Reference	Rationale for exclusion
Zucchelli G, Bernardi F, Montebugnoli L,De SM [37].	Smoker patients included (< 20 cigarettes/day)
Windisch P, Sculean A, Klein F, et al. [38].	Smoker patients included
Sculean A, Windisch P, Chiantella GC, et al. [45].	Smoker patients included
Minabe M, Kodama T, Kogou T, et al. [39].	Smoker patients included (< 10 cigarettes/day)
Meyle J, Gonzales JR, Bödeker RH, et al. [40].	Smoker patients included (< 20 cigarettes/day)
Sanz M, Tonetti MS, Zabalegui I, et al. [41].	Smoker patients included (< 20 cigarettes/day)
Parashis A, Andronikaki-Faldami A, Tsiklakis K [42].	Smokers patients included
Jepsen J, Heinz BB, Jepse Kn, et al. [43].	Smoker patients included (< 20 cigarettes/day)
Hoffmann T, Richter S, Meyle J, et al. [44].	Smoker patients included (< 20 cigarettes/day)
Röllke L, Schacher B, Wohlfeil M, et al. [46].	Patients > 18 years included
Silvestri M, Ricci G, Rasperini G, Sartori S, Cattaneo V. [47].	Patients > 21 years included
Silvestri M, Sartori S, Rasperini G, et al. [48].	Patients > 21 years included
Farina R, Simonelli A, Rizzi A, et al. [49].	Chronic or aggressive periodontitis patients included
Ghezzi C, Ferrantino L, Bernardini L, Lencioni M, Masiero S. [50].	Chronic or aggressive periodontitis patients included
Pontoriero R, Wennström J, Lindhe J. [51]	Outcomes not reported in terms of early wound healing
Jaiswal R, Deo V. [52]	No intervention treatment (EMD) present
Sculean A, Donos N, Miliauskaite A, Arweiler N, Brecx M. [55]	Report long-term data of a previous included study [56]
Sculean A, Schwarz F, Miliauskaite A, et al. [53].	Report long-term data of a previous included study [56]
Sculean A, Donos N, Schwarz F, et al. [54].	Report long-term data of a previous included study [45]

### Data analysis

To assess and detect similarities and differences between the studies - and to determine if it was possible to perform a further synthesis or comparison methods - data were summarized into evidence tables and a summary was performed.

Considerable heterogeneity was found in the studies, regarding the whole range of assessed parameters, including different follow-up time period evaluation, study design and specific GTR treatment. Moreover, the morphologic baseline characteristics of the surgical sites showed strong heterogeneity between groups of different studies, or it was often not reported. For this reason, it was not possible to conduct a reasonable data synthesis for the included studies and a meta-analysis could not be performed.

### Quality assessment and risk of bias assessment of selected publications

Six studies [56, 58–62] were randomized trials. According to the guidelines of the Cochrane Collaboration [34], three studies showed a high risk of bias [59, 60, 62] and three studies an unclear risk of bias [56, 58, 61] (Table 2).

**Table 2:** Summary of risk of bias of included RCTs

Domains	Adequate sequence generation?	Allocation concealment?	Blinding?	Incomplete outcome data addressed?	Free of selective reporting?	Free of other bias?
A.Sculean et al. 1999a [56]	Yes	Yes	Yes	Yes	Unclear	Unclear
A.Sculean et al. 1999b [60]	Unclear	Unclear	Yes	Yes	Unclear	No
N. Donos et al. 2004 [62]	Yes	Yes	Unclear	Yes	Unclear	No
A.Crea et al. 2008 [61]	Yes	Yes	Yes	Yes	Unclear	Yes
V. Iorio-Siciliano et al. 2011 [58]	Yes	Yes	Yes	Yes	Unclear	Yes
V. Iorio-Siciliano et al. 2014 [59]	Yes	Yes	No	Yes	Unclear	No

One study [57] was reported as a non-randomized clinical trial (a case-cohort study) and according to Cochrane Collaboration tool -ROBINS-I tool [35, 36], resulted to be a low risk of bias (Table 3).

**Table 3:** Summary of risk of bias of non-randomized clinical trial

Domains	Due to confounding	Selection of participants	Classification of interventions	Deviations from intended interventions	Missing data	Measurements of outcomes	Selection of the reported results
P. Cortellini et al. 2005 [57]	Low	Low	Low	Low	Low	Low	Low

### Description of the studies

#### Study characteristics

The characteristics of the studies are described in Table 4.

**Table 4:** Characteristics of included studies

Author, year of publication	Study design	Power calculation	Setting	Funding sources	Masking	Intervention	Follow-up
A.Sculean et al. 1999a [56]	RCT Split-mouth	No	U	Not specified	Double-blind	EMD vs BM	8 mo
A.Sculean et al. 1999b [60]	RCT Double-arm	No	U	Not specified	Double-blind	EMD vs BM	6 mo
N. Donos et al. 2004 [62]	RCT Multi-arm Three groups	No	U	Yes	Double-blind	EMD vs BM vs EMD + BM	12 mo
P. Cortellini et al. 2005 [57]	Non-RCT Case-cohort study Multi-arm Four groups	No	PP	Yes	Not performed	EMD vs BM vs BM + BG vs e-PTFE TrM	12 mo
A. Crea et al. 2008 [61]	RCT Double-arm	No	U	No	Double-blind	EMD vs e-PTFE M	36 mo
V. Iorio-Siciliano et al. 2011 [58]	RCT Double-arm	Yes	U	No	Double-blind	EMD vs e-PTFE TrM	12 mo
V. Iorio-Siciliano et al. 2014 [59]	RCT Double-arm	Yes	U	Not specified	Single-blind	EMD + DBBM vs BM + DBBM	12 mo

*BG* bone graft, *BM* bioabsorbable membrane, *DBBM* deproteinized bovine bone mineral, *EMD* enamel matrix derivative, *e-PTFE* expanded polytetrafluoroethylene; *mo* months, *M* membrane, *PP* private practice, *RCT* randomized clinical trial, *TrM* titanium reinforced membrane, *U* university

Four of the seven studies included in the present review were parallel (double-arm) studies [58–61], two [57, 62] were identified as multi-arm studies and one was designed as a split-mouth study [56].

A power calculation was performed in two of the seven studies [58, 59]. One study [57] was conducted in a private practice and the other six [56, 58–62] in university settings.

Regarding the funding sources, no according information was given in three of the studies [56, 59, 60]. For two of the studies [58, 61] no financial or material support was provided by any company. One study [62] reported industrial funding sources (Biora, Sweden and WL Gore). One other [57] was partly supported by scientific organizations (Accademia Toscana di Ricerca Odontostomatologica, Florence, Italy and the Periodontal Research fund of the Department of Periodontology of the Eastman Dental Institute, London U.K).

Five studies were double-blinded [56, 58, 60–62], while one was single-blinded [59] and in one study [57] no masking was performed.

Six different types of GTR techniques were compared with EMD: in four studies a bioabsorbable membrane was used [56, 57, 60, 62]. In two studies [57, 58] an expanded polytetrafluoroethylene (e-PTFE) membrane with titanium reinforcement, and in one study [58] without titanium reinforcement were used, while in two other studies the combination of a bioabsorbable membrane and bone graft [57] or bioabsorbable membrane and EMD [62] were selected. In one of the studies [59] EMD was not used as sole application but combined with deproteinized bovine bone mineral (DBBM) and compared with a control group, which employed DBBM and a collagen membrane [59]. Follow-up periods were reported at 6 months for one study [60], 8 months for one study [56], 12 months for four studies [57–59, 62] and 36 months for one study [61].

#### Population characteristics

##### Patient’s characteristics

A total of 199 patients with an age range between 30 and 73 years were assessed in the included studies. Two studies not reported the age of the patients [56, 60] and two studies not reported the gender [60, 62]. All patients enrolled in the studies [57–62] were explicitly reported to suffer from chronic periodontitis while in one study [56] the diagnosis was directly confirmed by the corresponding author to be chronic periodontitis (Table 5).

**Table 5:** Population characteristics

Author, year of publication	Patient’s characteristics					Teeth and defect characteristics at baseline	
	Number of patients	Gender (m/f)	Mean age/ Range (years)	Type of periodontitis	Drop-out	Number/Type of tooth	Number/Type of defects
A.Sculean et al. 1999a [56]	16	10 m/6f	NA NA	chronic periodontitis^a^	0	32 NA	32 2 to 3-wall intrabony defects
A.Sculean et al. 1999b [60]	14	NA	NA NA	chronic periodontitis	0	14 teeth scheduled for extraction	14 advanced intrabony defects (teeth scheduled for extraction)
N. Donos et al. 2004 [62]	9	NA	NA 40–73	chronic periodontitis	0	14 (EMD 4; GTR 3; EMD + GTR 7) mandibular molars	14 degree III furcation-involved defects
P. Cortellini et al. 2005 [57]	40	17 m/23f	41.3 ± 10.7 NA	chronic periodontitis	0	40 (e-PTFE TrM 12; BM + BG 11; BM 7; EMD 10) NA	40 intrabony defects 1-wall 1-wall 2 to 3-wall 3-wall
A. Crea et al. 2008 [61]	40	19 m/21f	45.8 35–66	chronic periodontitis	1	40 (39 evaluable) anterior/posterior	40 (39 evaluable) 3-wall intrabony defects
V. Iorio-Siciliano et al. 2011 [58]	40	19 m/21f	NA 39–52	chronic periodontitis	0	40 single-rooted teeth	40 non- contained intrabony defects combination ≥80% 1-wall component (2 to 3-wall component in the most apical part)
V. Iorio-Siciliano et al. 2014 [59]	40	18 m/22f	44.4 33–57	chronic periodontitis	0	40 single- and multi-rooted teeth	40 non- contained intrabony defects combination ≥70% 1-wall component (2 to 3-wall component in the most apical part)

*BG* bone graft, *BM* bioabsorbable membrane, *EMD* enamel matrix derivative, *e-PTFE* expanded polytetrafluoroethylene, *f* female, *GTR* guided tissue regeneration, *m* male, *NA* not available, *PD* probing depth, *TrM* titanium reinforced membrane

^a^confirmed by the author (A.S)

##### Teeth and defect characteristics at baseline

The studies reported 220 teeth with different intrabony and furcation defects (one defect per tooth); 97 defects were treated with EMD and 123 defects with GTR technique. In one study [62], degree III furcation-involved defects in mandibular molars were treated. In another study [61] 3-wall, angular intrabony defects in the interproximal area with an intrabony component ≥4 mm (measured from the crest to the deepest part of the bony defect) were selected. In one of the studies 2 to 3-wall defects were used [56] while in another [60] advanced intrabony defects (teeth scheduled for extraction) were treated.

Non-contained combined osseous defects in the interproximal area with an intrabony component ≥3 mm were treated in two of the studies [58, 59]. Finally, in one of the studies [57] different types of intrabony defects (1, 2 and 3 walls) were included and the treatment was assigned accordingly: 1-wall intrabony component 1–3 mm defects were treated with GTR with e-PTFE titanium reinforced membrane (TrM), 1-wall intrabony component 1–5 mm were treated with GTR (BM + BG) whereas in 2 to 3-wall narrow defects only MB was used. EMD was applied in the defects with a prevalent 3-wall component.

In one study the selected teeth were scheduled for extraction for periodontal or prosthetic reasons [60]. Two studies treated anterior and posterior teeth without furcation involvement [59, 61] whereas in another study [58] only single-rooted teeth including maxillary first premolars were selected. In two studies [56, 57] the type of tooth selected was not available (Table 5).

#### Treatment characteristics and early wound healing parameters assessed

Table 6 describes the main characteristics of the selected studies.

**Table 6:** Treatment characteristics and early wound healing assessment

Author, year of publication	Treatment prior to intervention	Intervention	Specific EMD treatment	Specific GTR treatment	Suture (material/time remotion)	Post-surgical medication	Periodontal parameters assessed	Maintenance	Parameters for early wound healing assessment
A.Sculean et al. 1999a [56]	3 mo bs: OhI + FM supra- and subgingival Sc	lA, intracrevicular incisions, full flap, GrTr, ScRp	2 min 24% EDTA gel, EMD	BM	NA 14 days	Amox (375 mg TID) Metro (275 mg TID) for 10 days	GI, BOP, PD, GR, CAL	0.12% CHX (TID) first 6 w. Tooth brushing resumed. Rv each 2 w (2 mo) and once a month afterwards	Allergic reactions, suppuration, abscess formation, swelling (1w). Membrane exposure (3w)
A.Sculean et al. 1999b [60]	3 mo bs: OhI + FM supra- and subgingival Sc	lA, intracrevicular incisions, full flap, GrTr, ScRp	2 min 24% EDTA gel, EMD	BM	Non-r e-PTFE sutures 14 days	Amox (1 g/d) for 1 w	PD, GR, CAL	0.12% CHX (BID) first 6 w. Tooth brushing resumed. Rv (professional tooth cleaning) each 2 w (6 mo)	Allergic reactions, suppuration abscess formation, membrane exposure
N. Donos et al. 2004 [62]	3 mo bs: OhI, ScRp	lA, intracrevicular incisions, full flap, GrTr, ScRp	2 min 24% EDTA gel, EMD (4 sites)	BM alone or BM + EMD	Non-r e-PTFE sutures 14 days	Metro (250 mg TID) for 1 w	BOP, PAL-V, PAL-H	0.2% CHX (BID) for 1 min first 6 w. Rv each 1 w (6 w): tooth polishing and Li 0.2% CHX. Tooth brushing resumed. Supragingival tooth polishing + OhI once a mo afterwards	Allergic reaction, abscess formation, membrane exposure (first 2 w)
P. Cortellini et al. 2005 [57]	Motivation, OhI, ScRp, Flap surgery in the remaining portions of the dentition	lA, SPPF, MPPT, crestal incision, full flap, GrTr, ScRp	EMD	e-PTFE TrM or BM or BM + BG	5–0, 6–0 and 7–0 Non–r e-PTFE sutures 7 days	Doxycycline (100 mg BID) for 1 week.	FMPS, FMBS, BOP, PD, GR, CAL, Rx defect angle	0.12% CHX (TID) and weekly prophylaxis for 6 w. Resumption oral hygiene:2 to 4 w after M removal or when BM were fully resorbed, after 4–5 w EMD group Rv monthly for 1 year	Primary closure recorded weekly for 6 w
A. Crea et al. 2008 [61]	3 mo bs: non-surgical periodontal therapy	lA, SPPF, full flap, periosteal releasing, GrTr, ScRp	2 min 24% EDTA gel, EMD	e-PTFE M	4–0 Non-r e-PTFE sutures 10 days	One day prior to surgery, Amox (500 mg BID) for 6 days	PD, CAL, GR, BOP, PI Is: IC, Rs-BC, CEJ-BD, CEJ-BC RMDD, RVBG	1% CHX gel (TID) (4 w) Rv each 1 w (first 6 w). Rv every 3 mo. *At each visit:* supra-gingival debridement teeth polish, OhI, BOP, PI assessment	Wound dehiscence, pain or discomfort, abscess formation, swelling, allergic reactions (5–6 days)
V. Iorio-Siciliano et al. 2011 [58]	Non-surgical mechanical debridement	lA, MPPT or SPPF,full flap, GrTr, ScRp	2 min 24% EDTA gel, EMD	e-PTFE TrM	5–0 Non-r sutures 7–10 days	600 mg Ibuprofen immediately before the surgery and after 4 h	FMPS, FMBS, PD, REC, CAL, Is: IVLD: CEJ-BD, VLD: BC-BD, HLD: Rs-BC, Rx defect angle	0.12% CHX (first 2 w) Modified oral hygiene procedures (4 w). Professional maintenance care after 2 and 4 w and after 3,6,9,12 mo	Early wound healing complications, membrane exposure after 1 w
V. Iorio-Siciliano et al. 2014 [59]	Non-surgical mechanical debridement	lA, MPPT or SPPF, full flap, GrTr, scaling and root planing	2 min 24% EDTA gel + DBBM particles (0.25 to 1.0 mm) + EMD	DBBM + BM	5–0 Non-r sutures 7–10 days	600 mg Ibuprofen immediately before surgery and after 4 h or 500 mg Acetaminophen immediately before and after 6 h	FMPS, FMBS, PD, REC, CAL, Is: CEJ-BD, VLD: BC-BD HLD: Rs-BC	0.12% CHX (first 2 w) Modified oral hygiene procedures (4 w). Professioal maintenace care after 2 and 4 w and after 3,6,9,12 mo	Early wound healing complications, membrane exposure after 1 w

Amox amoxicillin (systemic administration); *BC* bone crest, *BD* bottom of the defect, *BG* bone graft, *BID* twice times a day, *BM* bioabsorbable membrane, *BOP* bleeding on probing, *Bs* before surgery, *CAL* clinical attachment level, *CEJ* cemento-enamel junction, *CHX* chlorhexidine, *DBBM* deproteinized bovine bone mineral, *EDTA* Ethylenediaminetetraacetic acid, *EMD* enamel matrix derivative, *e-PTFE* expanded polytetrafluoroethylene, *FM* full mouth, *FMBS* full mouth bleeding score, *FMPS* full mouth plaque score, *GI* gingival index, *GR* gingival recession, *GrTr* granulation tissue remotion, *GTR* guided tissue regeneration, *HLD* horizontal linear distance, *IC* intrabony component of the defect, *Is* intrasurgical, *IVLD* intrasurgical vertical linear distance, *La* local anesthesia, *Li* local irrigation, *M* membrane, *mo* months, *min* minutes, *Metro* metronidazole, *MPPT* modified papilla preservation technique, *NA* not available, *Non-r* non resorbable, *OhI* oral hygiene instructions, *PAL-H* probing attachment level (mm) in the horizontal direction, *PAL-V* probing attachment level (mm) in the vertical direction, *PD* probing depth, *PI* plaque index, *RMDD* radiographic measurement of defect depth, *RVBG* radiographic vertical bone gain, *Rs* root surface, *Rv* recall visitis, *Rx* radiographical, *Sc* scaling, *ScRp* scaling and root planning, *SPPF* simplified papilla preservation flap, *VLD* vertical linear distance, *TID* three times a day, *TrM* titanium reinforced membrane, *w* weeks

##### Treatment prior to the intervention

In all the studies non-surgical periodontal therapy was performed. In four of them the pre-treatment initiated 3 months before surgery [56, 60–62], in the others no such time period was reported. In one study [60] - in which teeth scheduled for later extraction were selected as surgical sites - teeth were splinted before to reduce the mobility. One investigation also reported that non study-specific flap surgery was performed also in the dentition [57].

##### Intervention and specific treatment

The surgical procedures were similar in all the studies. The main difference was found to be the design of the incision. Local anaesthesia, full flap elevation, granulation tissue removal and scaling and root planing were described as common steps in all the studies. In three studies, intracrevicular incisions were performed [56, 60, 62] whereas in other three the simplified papilla preservation flap (SPPF) or modified papilla preservation flap (MPPT) [57–59] were selected according to the surgical site characteristics. In one of them [61] only SPPF was used.

The specific treatment on the EMD group consisted of a 2-min application of 24% EDTA gel and the application of EMD after careful rinsing for all studies. In one study EMD was combined with DBBM [59].

Regarding the specific treatment on the GTR group, in five of them a bioabsorbable membrane was used either alone [56, 57, 60, 62] or combined with EMD [62] or bone graft [57, 59]. In three studies [57, 58, 61] e-PTFE membrane was selected, in two of them with titanium reinforcement [57, 58].

In all studies non-resorbable suture materials were used (e-PTFE sutures were reported in four studies) [57, 60–62] except in one study [56] in which this data was not available. The time of suture removal was 14 days for three studies [56, 60, 62] and between 7 and 10 days for the rest of the studies [57–59, 61].

##### Post-surgical medication and maintenance

The post-surgical medication was different for all the studies. In three of them [56, 60, 61] amoxicillin was selected but the prescription was diverse, and in one of them was combined with metronidazole [56]. In one study [62] only metronidazole was indicated and in other doxycycline [57]. In two studies [58, 59] only anti-inflammatory drugs were used (ibuprofen or acetaminophen).

The antibiotic period of administration was between 1 week and 10 days and the anti-inflammatory drugs were indicated only the day of the surgical procedure.

Post-surgical chlorhexidine (CHX) was indicated in all the studies. In five studies 0.12% CHX was used for 2 [58, 59] and 6 weeks [56, 57, 60]. In one study [62] 0,2% CHX was indicated and also was complemented with weekly professional local irrigations for the first post-surgical 6 weeks. Finally, in one study [61] 1% CHX gel for 4 weeks was selected.

The maintenance period was similar in three studies in which recall visits were performed each 1 week for the first 6 weeks and then once a month [57, 62] or every 3 months [61]. In two studies [58, 59] the professional maintenance care was performed at 2 and 4 weeks and each 3 months afterwards. In two studies visits were scheduled every 2 weeks for all the follow-up time [60] or for the first 2 months and once a month afterwards [56]. The period necessary to resume oral hygiene procedures was also reported: in 3 studies [56, 60, 61] normal hygiene was initiated after 6 weeks, in two studies [58, 59] modified oral hygiene procedures were indicated for the first 4 weeks and in one study [57] was resumed 2–4 weeks after removal of the non resorbable membrane or after 4 weeks when resorbable membrane or EMD were used. In one study [61] this data was not clear.

##### Periodontal surrogate parameters

Clinical attachment level (CAL), probing depth (PD) and gingival recession (GR) were evaluated in all of the studies except in one of them [62]. Bleeding on probing (BOP) was evaluated in four of the studies [56, 57, 61, 62] whereas in three studies [57–59] full mouth plaque score (FMPS) and full mouth bleeding score (FMBS) were registered. Gingival index (GI) was registered in one study [56], and plaque index (PI) also was measured in only one study [61].

Intra-surgical and radiographic measurements of the defects were performed in three studies [58, 59, 61].

##### Early wound healing parameters assessed

No study reported data on EWH. Membrane exposure was evaluated in five studies [56, 58–60, 62]; in one study [56] this evaluation was performed at 3 weeks, in two studies at 1 week [58, 59], in one study [62] during the first 2 weeks whereas in one study [60] the evaluation time was not available.

Wound dehiscence was registered in two studies at the first week [61] or every week for the first 6 weeks [57].

Abscess formation was registered in four studies [56, 60–62], in two of them at the first week [56, 61]. The time was not reported for the other two studies [60, 62].

Suppuration was evaluated only in two studies [56, 60]. Pain/discomfort was registered by only one study [61] in the first 5–6 days.

Allergic reaction was evaluated in four studies [56, 60–62] and swelling was evaluated in two of them [56, 61].

### Early wound healing outcomes

Table 7 illustrates the early wound healing outcomes of the seven included studies.

**Table 7:** Early wound healing outcomes

Author, year of publication	Primary outcomes			Secondary outcomes		
	Flap dehiscence	Membrane exposure (GTR treated sites)	Suppuration	Abscess formation	Swelling	Allergic reaction
A. Sculean et al. 1999a [56]	–	7/16 GTR sites (3 w)	No	No	7/16 EMD sites 8/16 GTR sites (first w)	No
A. Sculean et al. 1999b [60]	–	No	No	No	–	No
N. Donos et al. 2004 [62]	–	2/3 GTR sites (BM alone), 5/7 GTR sites (BM + EMD) (first 2 w)	–	No	–	No
P. Cortellini et al. 2005 [57]	2/11 GTR sites (BM + BG), 1/7 GTR sites (BM alone) 1/10 EMD sites (1–2 w)	–	–	–	–	–
A. Crea et al. 2008 [61]	3/20 GTR sites 2/19 EMD sites (5–6 days)	–	–	No	No	No
V. Iorio-Siciliano et al. 2011 [58]	–	3/20 GTR sites (5 w)	–	–	–	–
V. Iorio-Siciliano et al. 2014 [59]	–	4/20 GTR sites (1 w)	–	–	–	–

*BM* bioabsorbable membrane, *BG* bone graft, *EMD* enamel matrix derivate, *GTR* guided tissue regeneration, *w* week

Flap dehiscence was evaluated in two studies [57, 61] and 79 sites. Dehiscences were observed in 6/50 (12%) of the GTR treated sites and in 3/29 (10.3%) of the EMD treated sites. Membrane exposure was evaluated in five studies [56, 58–60, 62] and was registered in 21/73 (28.8%) of the defects. In one of these studies [56], in which 7 of the 16 (43.7%) of the GTR treated defects showed exposition of the membrane at 3 weeks, swelling also was observed in 8 of the 16 sites at the first post-surgical week. Flap dehiscence, however, was not registered in sites treated with EMD while swelling were found in the same number of cases.

When flap dehiscence and membrane exposure were evaluated together it was observed that flap dehiscence was registered only 3.1% in the EMD treated sites whereas flap dehiscence/ membrane exposure was observed in the 22% of defects treated with GTR.

In all the remaining studies none of the others parameters evaluated (suppuration, abscess formation and/or allergic reaction) were observed. In only one study [60] the early wound healing was reported to be uneventful.

#### Healing outcomes associated to treatment characteristics

- Defect morphology

When 2 to 3-wall contained intrabony defects [56, 57, 61] were evaluated flap dehiscence/membrane exposure was presented in 11/42 (26%) sites treated by GTR and in 3/46 (6.5%) of sites treated with EMD. No dehiscence was observed when non-contained intrabony defects were treated with EMD (40 treated defects) [58, 59]. However, when GTR procedure was performed in these defects dehiscence/membrane exposure was observed in 9/63 (14%) of the treated sites [57–59].

One of the study [60] – in which advanced intrabony defects in teeth that were scheduled for extraction were assessed, did not report any complication in the healing process of neither group (EMD and GTR).

Finally, in the furcation GIII defects [62], 7/10 (70%) of the GTR treated sites presented membrane exposure while dehiscence was not observed in the EMD group (4 treated sites).

- Incision and flap design technique

When the incision/flap design was evaluated, membrane exposure was observed in 14/33 (42.4%) of the defects treated with GTR without any papilla preservation technique [56, 60, 62]. However, when SPPF or MPPT were used [57–59, 61] flap dehiscence was registered in 3/29 (10.3%) of the defects treated with EMD [57, 61] and flap dehiscence/ membrane exposure in 13/90 (14.4%) of sites treated with GTR (Table 6).

- Biomaterials

In cases treated with GTR, flap dehiscence/membrane exposure was found in 21/71 (30%) of the sites where resorbable membranes have been used [56, 57, 59, 60, 62] and in 6/52 (11.5%) of defects treated with non-resorbable e-PTFE membranes [57, 58, 61].

- Post-surgical medication

In two of the studies [58, 59] in which no antibiotics but only anti-inflammatory drugs were administered, early post-surgical complications were observed in 7/40 (17.5%) of the GTR sites whereas no complications were observed in the EMD treatment group.

When antibiotics were administered [56, 57, 60–62], complications like membrane exposure/dehiscence and swelling were observed in 21/83 (25%) of the GTR sites and in 10/56 (17.8%) of the defects treated with EMD.

- Suture

In three studies [56, 60, 62] in which the suture was removed after 14 days the percentage of sites with membrane exposure/flap dehiscence in the GTR defects was 42.4% (14/33) whereas in the EMD group 25.9% (7/27) of the sites presented post-surgical complications in terms of swelling as registered in one study [56].

In the remaining studies [57–59, 61] with a shorter suture removal time (7–10 days) 13.4% (16/119) of the treated sites showed dehiscence/membrane exposure. Moreover, when this was analysed separately for the treatment groups it was found that the complications were reported in 14.4% of the GTR group and 5% of the EMD sites.

In total, of 219 evaluated sites flap dehiscence /membrane exposure was registered in 22% (27/123) of the defects treated with GTR versus 3.1% (3/96) in the EMD group.

If all the parameters evaluated are grouped as post-surgical complications, complications were observed in 28/123 (22.8%) of the GTR sites and in 10/96 (10.4%) of the sites treated with EMD.

## Discussion

Wound closure is one of the most important factors in obtaining successful clinical results, especially in regeneration procedures [18]. With this regard, the first post-operative week has been considered critical for the maintenance of wound stability [19].

Findings from human studies have indicated that EMD may play a major role in periodontal wound healing in terms of fewer post-surgical complications when compared to GTR surgical techniques and improved healing of incisions by promoting formation of blood vessels and collagen fibers in the connective tissue [29]. Moreover, clinical studies have indicated that treatment with EMD positively influences periodontal wound healing after surgical treatment [17]. However, another clinical study showed that the early wound healing of periodontal flap-surgeries in the sites treated with EMD was not different from control sites which were treated by open flap debridement alone [63].

The present systematic review was performed to evaluate whether or not the use of EMD in regenerative surgical treatment of periodontal intrabony defects show better results in terms of early wound healing when compared to GTR treatment.

The primary outcome parameters were registered between one and six post-surgical weeks. In this regard, seven studies could be compared. Due to a strong heterogeneity a meta-analysis could not be performed, but a descriptive data analysis revealed clinically relevant findings.

First, the data suggest that there is no relevant difference in the early wound healing outcomes between the two treatments evaluated, since flap dehiscence was observed in the 12% of the GTR treated sites and in the 10.3% of the EMD treated sites [57, 61]. Second, other parameters as suppuration, abscess and allergic reactions were not reported in any of the studies. Swelling was reported in one study [56] but with no difference between the two treatment groups. However, membrane exposure was observed in the 28.8% of the GTR treated sites in 5 studies [56, 58–60, 62]. While this finding was reported in a considerable number of times, the control group using EMD did not show such undesired wound healing. In our reading the phenomenon “membrane exposition” is strictly related to flap dehiscence. A flap dehiscence may not necessarily result always in a membrane exposure but if a membrane exposure is present, it means that a dehiscence of the flap has also occurred. Therefore, this parameter should not be considered separately. Moreover, none of the studies included reported both parameters. Dehiscence was evaluated in only two of the studies [57, 61] while membrane exposure in five of them [56, 58–60, 62]. Therefore, an analysis of both parameters together could be useful.

If we consider this analysis and match the information resulting of both parameters, we can observe that flap dehiscence was registered in a minimal amount (3.1%) in the EMD treated sites whereas flap dehiscence/ membrane exposure was observed in the 22% of GTR treated defects. This is in agreement with a previous multicentre study in which more post-surgical complications following GTR were observed as compared to sites treated with EMD [28]. According to everything mentioned above, we remain with our null hypothesis neither confirmed nor rejected.

In second place, as complete primary wound closure of the flap during early wound healing is a prerequisite for the success of regenerative therapy [64], the following factors should be considered [65]: 1) incision and flap design techniques; 2) correct suture technique and removal time; 3) adequate post-surgical controls and maintenance therapy; 4) type of biomaterials used.

### Incision and flap design techniques

It has been reported that the use of inter-dental tissue preservation surgical techniques provides a better flap stabilization [66]. It is important to note that in the three studies [56, 60, 62] where intracrevicular incisions were made without any interdental surgical preservation technique, membrane exposure was observed in almost half of the GTR of the treated defects (42.4%); conversely, when SPPF or MPPT were used [57–59, 61] flap dehiscence/membrane exposure was registered in only 10.3% of the EMD treated defects [57, 61] and in the 14.4% of the GTR treated sites (Table 6).

### Suture technique and removal time

Suturing is one of the most important factors related to wound stability [67], especially during the first post-surgical weeks when adherence of the flap to the underlying hard tissues is only guaranteed by a thin blood clot that is converting to fibrous and osseous tissue [68, 69]. In fact, in regenerative procedures the suture is normally removed after 10 to 14 days post-surgery [68, 70]. It has been demonstrated that the use of thick sutures (4–0) and/or early suture removal can result in dehiscences of the formerly adapted flaps [71]. In the studies included in the present review this was not explicitly reported, even in three studies [56, 60, 62] in which the suture was removed at 14 days the percentage of sites with membrane exposure was higher (42.4%) as compared to studies where this time was shorter (7–10 days), with only 13.4% of the treated sites with dehiscence/membrane exposure [57–59, 61] (Table 6).

### Post-surgical indications and maintenance

The post-surgical controls and maintenance therapy have also been evaluated in this article. In general, the first follow-up visit was scheduled 1 week after surgery [72] and, in regenerative therapies with membranes, the recall visits turned out to be more frequent during the first 2–3 weeks, when professional tooth cleaning was performed [67, 73]. In all evaluated studies the first post-surgical control was performed at one post-surgical week and the recall visits were indicated every 1 or 2 weeks for the first 6–8 weeks. In one study [61] the first evaluation was made at 5 days and, at this time, a wound dehiscence could be observed in the 14% (5/39) of the treated sites. This aspect is of paramount importance because early controls might help to detect early complications as can be a small flap dehiscence without a membrane exposure. Moreover, the time to resume hygiene oral procedures must be considered since it has been reported that only after 4–5 weeks the flap is completely reattached to teeth and bone [65, 68]. This period was considered in all the studies and the oral hygiene procedures were resumed between 4 to 6 post-surgical weeks (Table 6).

### Biomaterials

It has been demonstrated that non-resorbable membranes have a higher risk of exposure than resorbable membranes in GTR procedures [74]. In the included studies, no such effect was shown. Of 123 sites treated by GTR, in 71 resorbable membranes were used and in 52 e-PTFE membranes. Surprisingly, flap dehiscence/membrane exposure was present in 30% of the sites treated with resorbable membranes, whereas only in 11.5% sites with non resorbable e-PTFE membranes (Table 6). Regarding this point, it is important to underline that in the selected studies 20 of the 52 treated sites were 3-wall contained intrabony defects. Studies [11, 75, 76] show a good prognosis when these defect types are treated with others surgical approaches or with resorbable membranes.

It is important to highlight the fact that, when all the previous mentioned factors related with the early wound healing were evaluated, the group treated with EMD presented a lower percentage of sites with post-surgical complications respect the GTR group. This could be related to the aforementioned properties of the EMD in the wound healing [15, 31].

In fact, if both evaluated parameters (dehiscence and membrane exposure) are considered as one kind of post-surgical complications, complications were observed less often after EMD procedures than after GTR procedures. This is in agreement with the results observed in a systematic review [15] and clinical studies [41] in which more post-surgical complications following GTR than after EMD application have been reported. In fact, in a multicentre clinical trial [41]in which 75 patients were treated, it was observed that all cases treated with GTR presented a post-surgical complication, mostly membrane exposure, while only 6% of EMD treated defects showed complications. This study was not included in the present revision since smokers were also evaluated.

Another important parameter is the administration of systemic medications and especially antibiotics after or during the surgical procedures. While a few studies concluded that better healing and less discomfort is observed when antibiotics were given [77, 78], in many other studies [79–82] the use of antibiotics was considered not necessary. Although there is currently no consensus regarding this aspect, this parameter was assessed in the present review (Table 6) in order to avoid a possible bias but, given that in all studies drugs and posology were vastly different, establishing any conclusion from the given data seems inappropriate. However, it was observed that in two of the studies [58, 59] in which antibiotics were not administered but only anti-inflammatory drugs (Ibuprofen or Acetaminophen), the early post-surgical complications (17.5% GTR group and 0% EMD group) were not more frequent than in the studies with antibiotic administration. In fact, the complications registered were even more frequent in the “antibiotic group” [56, 57, 60–62], in which 25% of the GTR and 17.8% of the EMD group presented membrane exposure/dehiscence and/or swelling. This is coincident with a previous clinical study [82] that evaluated the role of antibiotics in preventing early post-operative complications after periodontal surgical procedures. The evaluation was performed 1, 2, 4, 7 days and 3 months after surgery and 3 groups were evaluated (amoxicillin, doxycycline and no antibiotics). The authors reported no differences in terms of early complications between the three groups. They concluded that performing the surgical procedures following strict asepsis the prevalence of complications is low. Accordingly, prophylactic antibiotic to prevent post-operative complications was considered unnecessary.

Two important aspects related to the included studies should be especially considered: first, the already mentioned heterogeneity observed among all the studies and especially for defect morphology and second the studies ‘quality.

With respect to the first point, one of the most notable differences between the studies was the morphology of the defects (Table 5). Although generally in all studies intrabony defects were included, the spectrum ranged from “advanced intrabony defects” (scheduled for extraction) over 3-wall defects, partially non-containing defects and GIII furcation defects. However, a descriptive analysis distinguishes the different types of defects with respect to the individual treatment that was performed.

In fact, the defect morphology –related also with the surgical approach and the biomaterials selected - strongly influences the results of the surgical procedures [11, 57, 75, 76]. Clinical success was reported when contained intrabony defects were treated with EMD. In non-contained intrabony defects, GTR procedures are more indicated [11, 75, 76] although it has been observed in a recent study successful clinical results when non-contained intrabony defects were treated with EMD [83].

In the present systematic review, when the 2–3 wall contained intrabony defects were evaluated it was observed flap dehiscence/membrane exposure in 26% of the sites treated by GTR whereas flap dehiscence was observed in only 6.5% of the sites treated with EMD [56, 57, 61]. Instead, in non-contained defects [57–59] 14% of the GTR treated sites showed membrane exposure while no post-surgical complications were observed in the EMD group.

Finally, in the study treating furcation III defects [62], membrane exposure was observed in seven of the ten treated sites (70%). This study was the only one that compared EMD with either GTR or GTR + EMD. When the membrane exposure was assessed in the GTR groups 2 (67%, with GTR) of 3 and 5 (71%, with GTR + EMD) of 7 sites were find to show that. Although no meta-analysis was performed due to the small power of the published data it seems that EMD as an adjunct to GTR did not provide an additional benefit in the treatment of furcation GIII defects. At the final follow-up, the results also demonstrated that only a partial closure of the furcation entrance was achieved. This finding was in accordance with a previous clinical study [84] which could not be considered in this review since smokers were included.

Regarding the quality of the included studies [34–36], the present review included six RCTs and one non-randomized clinical trial (a case-cohort study) comparing EMD and GTR surgical procedures. The quality of the  RCTs were found to be moderate to low, considering that three studies showed a high risk of bias [59, 60, 62] and three studies an unclear risk of bias [56, 58, 61] (Table 2). The only study reported as non-RCT [57] resulted to have a low risk of bias (i.e., the study is comparable to a well-performed randomized trial with regard to all the domains; Table 3). Furthermore, a potential bias regarding the funding sources has to be considered. In concerning this matter there was only one study included that was supported by external companies that sponsored the different biomaterials used for both study groups (EMD and GTR) [62], what rendered the risk for bias rather low.

In the present systematic review, we decided to exclude smokers to avoid possible bias considering that smoking affects the wound healing process [85]. Indeed, in a recent clinical study [86] in which the impact of smoking status on the clinical outcomes after regenerative surgical procedures were evaluated, the authors concluded that in smoker patients wound healing quality was significantly hampered when compared to non-smokers. A dose-dependent effect of smoking was observed with respect to the values of PD reduction and CAL gain at 6 months with a tendency to lower values in patients consuming 11–20 cigarettes/day than in smokers from 1 to 10 cigarettes/day. Accordingly, even light smokers were excluded from the present analysis.

Specifically assessing early wound healing outcomes, there are no RCTs comparing EMD and GTR for the treatment of intrabony defects. Most of the studies focus however on the long-term clinical outcomes after 12 months [76]. In addition, in none of the studies included in the present revision, early wound healing was evaluated with any of the indices/systems already proposed in the literature [23, 24, 86].

Within the present systematic review, no relevant differences in the early wound healing results between EMD and GTR surgical treatment in periodontal intrabony defects can be found, although - when a deeper and detailed evaluation of the studies was performed - a tendency for better early healing in the group treated with EMD seems evident. Particularly, when the analysis was performed considering the different defect types it was observed that both contained and non-contained intrabony defects presented a higher percentage of dehiscence/membrane exposure when GTR treatment was performed. These findings however should be interpreted with care given the heterogeneity and the quality of the studies included. The higher risk for dehiscence and membrane exposure in GTR procedures, however, cannot be interpreted as general superiority of EMD in the early wound healing of the treatment of intrabony defects. In fact, when only flap dehiscence was analysed the results observed were similar for both treatment groups (12% GTR versus 10.3% EMD treated sites).

Therefore, future RCTs comparing EMD and GTR surgical procedures in terms of early wound healing are necessary to understand if EMD presents an additional benefit in this regard. In order to render a quantitative meta-analysis possible study designs should be standardized to reduce heterogeneity and possible biases. Moreover, long-term studies that compare early wound healing outcomes to the final results would allow to deepen the insight into the effect of uneventfully early healing.

Finally, it is important to mention that the purpose of the present systematic review is not to suggest the one or the other treatment type. Clinically, the decision for/against EMD/GTR is multifactorial [76] and depends especially on the defect morphology. Furthermore, the number of sites to be treated and their localization might be of relevance, since multiple defects or defects difficult to reach might easier and less expensively be treated with EMD due to a quicker and easier application as compared to the GTR protocol.

## Conclusion

Due to the considerable heterogeneity of the published studies, a clear beneficial effect of the EMD on the early wound healing outcomes after surgical treatment of periodontal intrabony defects cannot be confirmed.

Standardized RCT studies are needed in order to allow for proper comparison of early wound healing after both types of surgical approaches.

## Abbreviations

BOP: Bleeding on probing
CAL: Clinical attachment level
CHX: Chlorhexidine
CTs: Clinical trials
DBBM: Deproteinized bovine bone mineral
EMD: Enamel matrix derivatives
e-PTFE: Expanded polytetrafluoroethylene
EWH: Early wound healing index
FMBS: Full mouth bleeding score
FMPS: Full mouth plaque score
GI: Gingival index
GR: Gingival recession
GTR: Guided tissue regeneration
MPPT: Modified papilla preservation technique
PD: Probing depth
PGA: Propylene glycol alginate
PI: Plaque index
RCTs: Randomized clinical trials (RCTs)
SPPF: Simplified papilla preservation flap
